# *Cinnamomi Ramulus* inhibits the growth of colon cancer cells via Akt/ERK signaling pathways

**DOI:** 10.1186/s13020-022-00588-6

**Published:** 2022-03-09

**Authors:** Boyu Pan, Yafei Xia, Zilu Gao, Gang Zhao, Liangjiao Wang, Senbiao Fang, Liren Liu, Shu Yan

**Affiliations:** 1 Tianjin Key Laboratory of Acute Abdomen Disease Associated Organ Injury and ITCWM Repair, ITCWM Hospital, Tianjin University, No.92 Weijin Road, Nankai District, Tianjin, 300072 China; 2 Department of Gastrointestinal Cancer Biology, Tianjin Medical University Cancer Institute & Hospital, Key Laboratory of Cancer Prevention and Therapy, Tianjin; Tianjin’s Clinical Research Center for Cancer, National Clinical Research Center for Cancer, Huanhuxi Road, Hexi District, Tianjin, 300060 China; 3 Department of Maxillofacial and Otorhinolaryngological Oncology, Tianjin Medical University Cancer Institute & Hospital, Key Laboratory of Cancer Prevention and Therapy, Tianjin; Tianjin’s Clinical Research Center for Cancer, National Clinical Research Center for Cancer, Tianjin, 300060 China; 4 School of Information Science and Engineering, Central South University, Yuelu District, Changsha, 410006 Hunan China

**Keywords:** *Cinnamomi Ramulus* (CR), Colon cancer (CC), Chinese herb medicine (CHM), Network pharmacology, Bioinformatics, Akt/ERK signaling pathways

## Abstract

**Background:**

Colon cancer (CC) ranks the second highest mortality rate among malignant tumors worldwide, and the current mainstream treatment regimens are not very effective. The unique efficacy of Chinese herb medicine (CHM) for cancer has recently attracted increasing attention. *Cinnamomi Ramulus* (CR), as a classic CHM, has been widely used in the treatment of a variety of diseases for hundreds of years in China, but its specific pharmacological mechanism against CC needs to be fully evaluated.

**Methods:**

TCMSP and China National Knowledge Infrastructure database were utilized to predict the candidate ingredients of CR, and TCMSP and SwissTargetPrediction database were also employed to predict the drug targets of the candidate ingredients from CR. We subsequently evaluated the therapeutic effect of CR by orally administrating it on CC-bearing mice. Next, we further identified the potential CC-related targets by using Gene Expression Omnibus (GEO) database. Based on these obtained targets, the drug/disease-target PPI networks were constructed using Bisogenet plugin of Cytoscape. The potential core therapeutic targets were then identified through topological analysis using CytoNCA plugin. GO and KEGG enrichment analyses were performed to predict the underlying mechanism of CR against CC. Furthermore, these in silico analysis results were validated by a series of cellular functional and molecular biological assays. UPLC–MS/MS method and molecular docking analysis were employed to identify the potential key components from CR.

**Results:**

In this study, we firstly found that CR has potential therapeutic effect on cancer. Then, oral administration of CR could inhibit the growth of CC cells in C57BL/6 mice, while inhibiting the viability and motility of CC cells in vitro. We obtained 111 putative core therapeutic targets of CR. Subsequent enrichment analysis on these targets showed that CR could induce apoptosis and cell cycle arrest in CC cells by blocking Akt/ERK signaling pathways, which was further experimentally verified. We identified 5 key components from the crude extract of CR, among which taxifolin was found most likely to be the key active component against CC.

**Conclusions:**

Our results show that CR as well as its active component taxifolin holds great potential in treatment of CC.

**Supplementary Information:**

The online version contains supplementary material available at 10.1186/s13020-022-00588-6.

## Background

Colon cancer (CC) is currently one of the five major malignant tumors in the world. According to cancer statistics published by the American Cancer Society, the mortality rate of CC has reached 9.2%, increasing to second among all cancer types [1–3]. Currently, tumor resection, chemotherapy, radiofrequency ablation, and targeted therapy are still the mainstream treatment methods for CC [4]. However, the overall efficacy of these treatments is not satisfactory [5]. Therefore, there is an urgent need to find new treatments to improve the survival and prognosis of CC patients.

**Fig. 1:**
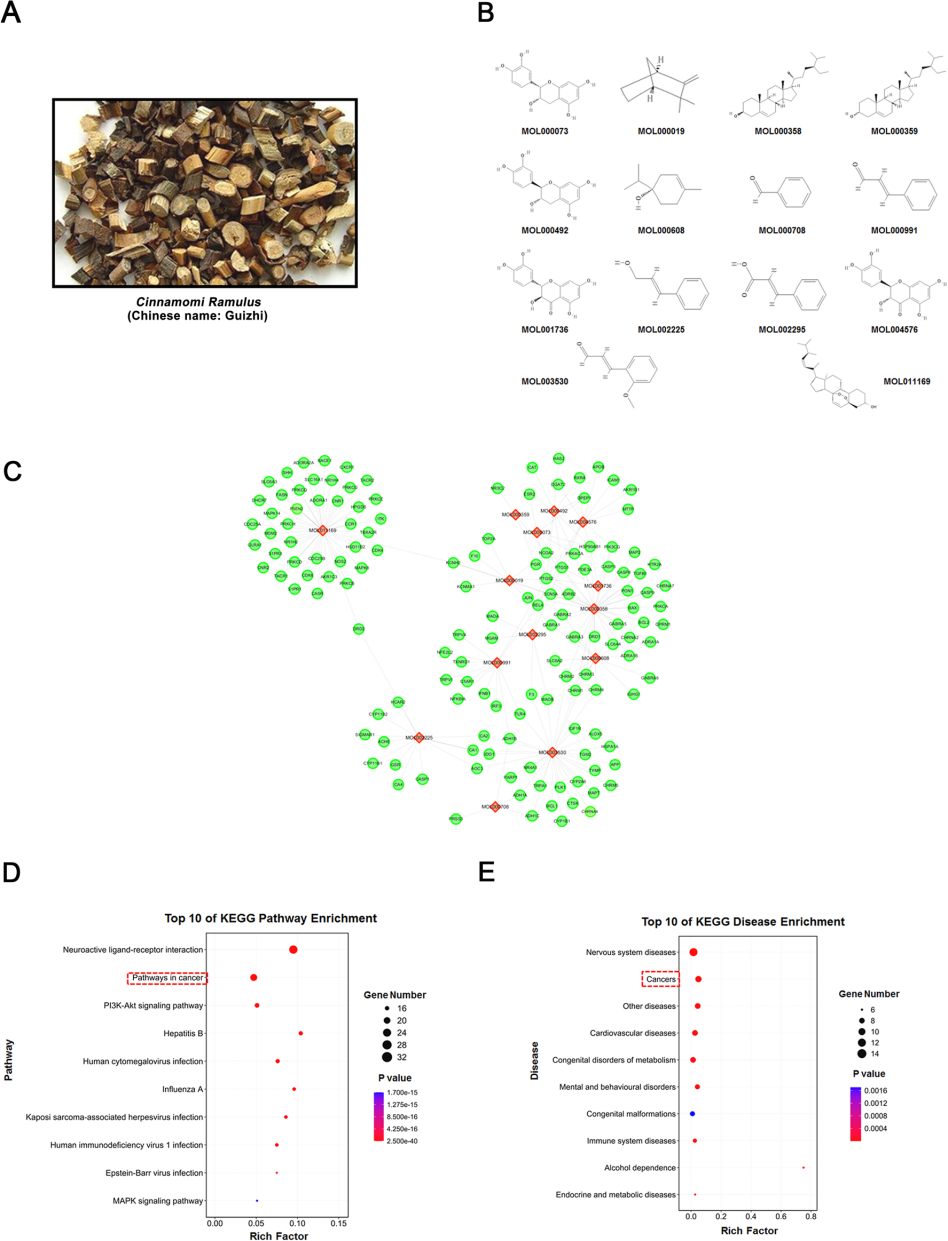
Construction of the CR compound-target systematic network and enrichment analyses of the putative targets. **A** The physical map of *CR*. **B** The 14 potential active compounds in *CR*. **C** The systematic network was constructed by linking the potential active compounds and their putative drug targets in *CR*. **D** and **E** Putative drug targets were enriched in the KEGG pathway and disease using DAVID v6.8 and KOBAS 3.0, respectively (*p* < 0.05)

As an important component of traditional Chinese medicine (TCM), Chinese herbal medicine (CHM) has been widely used in the treatment of various diseases in China for thousands of years. Recent studies have shown that CHM provides a key option for the treatment of complex diseases, including cancer, due to its unique clinical efficacy [6, 7]. *Cinnamomi Ramulus* (CR, Chinese name: Guizhi), the dried branch of cinnamon, is an important representative of CHM. It first appeared in China's classic Han dynasty medical treatise *Shang Han Lun* (Treatise on Cold Damage) (Fig. 1A). In TCM theory, CR induces sweating and relieves exterior syndromes, dissipates cold and relieves pain, promotes yang and transforms qi. Modern pharmacology has confirmed that CR has antibacterial, antiinflammatory, antiviral, hypoglycemic and antiplatelet agglutination effects [8]. Since 1973, CR has been listed as an important TCM by the *Chinese Pharmacopoeia* due to its definite efficacy, minimal toxic side effects, and stable quality. Additionally, more than 60 types of Chinese medicine preparations containing CR (e.g. Danggui Sini Decoction and Huangqi Guizhi Wuwu Decoction) have been included in the *Chinese Pharmacopoeia* [8]. Recent studies have shown that some TCM formulas containing CR have good antitumor effects. Dai et al. reported that Guizhi Fuling Decoction could play a role in the treatment of breast cancer by interfering with the phosphatidylinositol 3-kinase (PI3K) and mitogen-activated protein kinase (MAPK) signaling pathways [9]. Another study showed that Danggui Sini Decoction, a traditional classic recipe, could affect some key biological processes, such as apoptosis and the cell cycle, involved in the survival and growth of gastric cancer cells. The disruption of these biological processes may be attributed to the simultaneous inhibition of a variety of molecular signaling pathways, including the PI3K/Akt, MAPK, and p53 pathways [10].

In recent years, network pharmacology has gradually become a promising method for studying TCM from a systematic perspective [11]. The network pharmacology of TCM integrates pharmacology, phytochemistry, bioinformatics, and computational biology techniques. These methods help to study the pharmacological mechanism of TCM and bridge the gap between Western medicine and TCM [12, 13]. The results of several previous studies have shown that some components from CR have potential therapeutic effects on CC [14, 15]. However, the overall pharmacological mechanism of CR in anti-CC and its potential clinical application value need to be fully evaluated. Therefore, we conducted this study guided by TCM network pharmacology, bioinformatics, and molecular docking analysis. Through a series of in vivo and in vitro experiments, we demonstrated that CR can exert a significant direct inhibitory effect on the growth of CC cells by blocking the Akt/ERK signaling pathway.

## Methods

### Cell culture and related reagents

HT-29 and HCT-15 human colon carcinoma cells and MC-38 murine colon carcinoma cells were purchased from China Infrastructure of Cell Line Resources (China) and then cultured in DMEM and RPMI-160 medium, respectively, supplemented with 10% (v/v) fetal bovine serum (FBS) and 100 U/ml streptomycin/penicillin at 37 °C and 5% CO_2_. Cell Counting Kit-8 (CCK-8) reagent was purchased from Dojindo (Japan). Hoechst 33342 reagent was purchased from Beyotime (China). An Annexin-V FITC apoptosis detection kit (#556547) and FITC BrdU cycle detection kit Part A (#559619) were purchased from BD Biosciences PharMingen (USA). Taxifolin was purchased from Aladdin (China). Antibodies against Bcl-2, Bax, caspase 9/p35/p10, poly (ADP-ribose) polymerase-1 (PARP1), cyclin D1, and beta-actin were purchased from Proteintech. Antibodies against cyclin A2, cyclin B1, p-Akt (S473), p-Akt (T308), pan-Akt, p-ERK (T202/Y204) and pan-ERK were purchased from CST. Antibody against CDK2 was purchased from Abcam. CR powder was provided by the Chinese Medicine Pharmacy of Tianjin Medical University Cancer Institute and Hospital. The concentration of the prepared CR solution was 0.05 g/ml. In addition, for the subsequent relevant biological experiments, the 0.05 g/ml solution was subjected to 0.22 μm filter membrane filtration and sterilization and dilution to the necessary concentrations.

### Screening of potential active components in CR

The traditional Chinese medicine systems pharmacology database and analysis platform (TCMSP) is a database and visualization platform established based on the pharmacology framework of the CHM system (http://tcmspw.com/tcmsp.php). It includes 499 CHMs, 29,384 components, 3311 targets, and 837 related diseases registered in the *Chinese Pharmacopoeia* [16]. In this study, the main active compounds of CR were first searched through the TCMSP online platform, with “*Cinnamomi Ramulus*” as the key word, and then, all the compounds obtained in the search were then screened for potential activity based on absorption, distribution, metabolism, and elimination (ADME)-related parameters. The parameters were set as oral bioavailability (OB) ≥ 30% and drug-like activity (DL) ≥ 0.18, and the relevant active ingredients were obtained after screening. In addition, we also searched the China National Knowledge Infrastructure (www.CNKI.net) to obtain the main active components in CR; however, not all of the abovementioned parameters were met.

### Screening of potential drug targets corresponding to CR and disease targets closely related to CC

First, TCMSP and the SwissTargetPrediction database (http://www.swisstargetprediction.ch/) were used to query the drug targets corresponding to the potential active compounds of CR obtained via the abovementioned methodology. The UniProt online database (http://www.uniprot.org/) was used to query the gene name corresponding to each protein target, verify the species origin as "human" and create a dataset for subsequent analysis. Additionally, we obtained the known disease targets closely related to CC from the Gene Expression Omnibus (GEO) database (https://www.ncbi.nlm.nih.gov/geo/), which includes two gene expression dataset microarrays (GSE44076 and GSE13471) from human-derived CC and adjacent normal tissues. Then, the protein–protein interaction (PPI) data were analyzed using the core plugin Bisogenet in Cytoscape software. The final results were integrated into a single result for six independent analyses of the PPI data obtained above.

### Systematic network construction and enrichment analysis

We first constructed PPI network relationships between candidate drug targets of CR and disease targets associated with CC through the Bisogenet and visualized the images with Cytoscape software (Version 3.2.1) [17]. Then, the above 2 PPI networks were merged, and the topological parameters of each node in the merged network were calculated using CytoNCA, another Cytoscape plugin. In the merged network system, the nodes with twice the median of 'degree centrality' (DC) were considered important nodes, and then, these nodes were used to generate a new relational network. Next, to further screen the potential core targets of CR for the treatment of CC, we mined the topological parameters of the new network that were higher than the ‘DC’, ‘betweenness centrality’ (BC) and ‘closeness centrality’ (CC') medians. These targets were considered to be the most significant among all nodes. Then, we used KOBAS 3.0 (http://kobas.cbi.pku.edu.cn/), The Database for Annotation, Visualization and Integrated Discovery (DAVID) v 6.8 (https://david.ncifcrf.gov/) and Metascape (https://metascape.org/) to conduct the enrichment analysis of biological functions and signaling pathways for the obtained candidate drug targets and core targets.

### Mouse xenograft experiment

Four-week-old male C57BL/6 mice were purchased from Beijing Vital River Laboratory Animal Technology Co., Ltd. (China). All procedures involving animals were performed in accordance with the regulations of the Ethics Committee of the Hospital of Integrated Traditional Chinese Medicine and Western Medicine, Tianjin University (ITCWM Hospital, Tianjin University). Ten animals were randomly divided into 2 groups, with 5 animals in each group. At 5 weeks of age, MC-38 murine colon carcinoma cells (1.0 × 10^6^/100 μL) transfected with a luciferase reporter plasmid were subcutaneously injected into the right hip area of the 10 mice using a 1-ml needle. After 10 days, when tumors were visible to the naked eye, 200 µL of normal saline (blank control group) or CR solution (300 mg/kg body weight) was administered to the mice by gavage (po) twice daily during the experimental period (drug administration was performed in the morning/afternoon). The tumor volume was calculated once per day using the following formula: long diameter × (short diameter)^2^/2. On day 14, tumor-bearing mice were observed and photographed using a small animal imaging system. Then, all mice were sacrificed, and tumor tissue was weighed. None of the mice died before the end of the experimental period.

### Immunohistochemistry

Slides containing tumor tissue were deparaffinized, and antigen retrieval was performed. Then, the slides were incubated with anti-Ki-67 antibody (Abcam, UK) overnight at 4 °C. After washing with phosphate buffered saline (PBS), the slides were incubated with Polymer Helper and poly peroxidase-anti-mouse/rabbit IgG (PV-9000, ORIGENE, China) and then further incubated with diaminobenzidine (DAB).

### Cell function and molecular mechanism verifications

The procedures for assessing cell viability and plate colony formation were described previously [18, 19]. The cumulative motility of CC cells was calculated using an Operetta CLS™ high-content analysis system equipped with Harmony software (Perkin Elmer, Waltham, MA, USA). The measurement was performed in digital phase contrast mode at 37 °C and 5% CO_2_ using a × 20 long distance objective. In addition, apoptosis and cell cycle assays and western blots (WBs) were performed [18].

### High-performance liquid chromatography coupled with mass spectrometry and sample extraction

High-performance liquid chromatographic separation and mass spectrometric detection were performed using Waters Quattro Premier XE/Acquity UPLC system coupled to a tandem quadrupole mass spectrometer (Waters Corporation, Milford, MA, United States). Lab solutions LCMS software (Masslynx V4.1) was used to control the instruments and process the data. Please see details as we previously described [19].

### Molecular docking analysis

AutoDock software (v4.2) was used to dock the structures of five ingredients, including cinnamic acid, 2-Hydroxycinnamic acid, protocatechuic acid, catechinic acid and taxifolin in *Cinnamomi Ramulus* detected by UPLC-MS/MS. These ingredients were docked with PRKCA (PDB code: 4DNL), HSP90AB1 (PDB code: 1UYM), APP (Alphafold code: P05067), RELA (PDB code: 2O61), JUN (Alphafold code: P05412), MDM2 (PDB code: 1T4E), PRKACA (PDB code: 2GU8), HSPA1A (PDB code: 1HJO) and PARP1 (PDB code: 1UK0), respectively. Please see details as we previously described [10].

### Statistical analyses

All data were analyzed using SPSS 17.0 software (USA). Results were represented as mean with standard deviations (mean ± SD). The differences expressed were using the Student's *t*-test, and *p* < 0.05 was considered as statistically significant.

## Results

### Screening and enrichment analysis of candidate active components and potential drug targets of CR

To investigate the pharmacological action mechanism of CR, we first performed virtual screening based on the two important parameters of ADME, i.e., OB and DL, to obtain the potential active components in CR. Seven monomers met the requirements of OB ≥ 30% and DL ≥ 0.18. Then,we also identified seven other monomeric components that either showed good pharmacological activity or were typical main components of CR reported in the literature. They were included for subsequent analysis; however, for the components, either OB was less than 30% or DL was less than 0.18. Therefore, a total of 14 compounds were considered potential monomerically active components of CR (Fig. 1B and Table 1).

**Table 1:** The specific information of candidate active ingredients in CR obtained from a virtual screening and literature mining

MOL number	Candidate ingredients	OB/%	DL
MOL000019	D-Camphene	34.98	0.04
MOL000073	ent-Epicatechin	48.96	0.24
MOL000358	Beta-sitosterol	36.91	0.75
MOL000359	Sitosterol	36.91	0.75
MOL000492	( +)-Catechin	54.83	0.24
MOL000608	()-Terpinen-4-ol	81.41	0.03
MOL000708	Benzaldehyde	32.63	0.01
MOL000991	Cinnamaldehyde	31.99	0.02
MOL001736	(−)-Taxifolin	60.51	0.27
MOL002225	Styrone	38.35	0.02
MOL002295	Cinnamic acid	19.68	0.03
MOL003530	O-Methoxycinnamaldehyde	26.52	0.04
MOL004576	Ttaxifolin	57.84	0.27
MOL011169	Peroxyergosterol	44.39	0.82

Subsequently, we further used two online databases, i.e., TCMSP and SwissTargetPrediction, to explore the candidate drug targets of the above 14 compounds and obtained a total of 142 drug targets (Additional file 1: Table S1). Interestingly, there were many overlapping targets among the 14 different components, indicating that these compounds may play key roles in generating synergistic effects. Then, we further used Cytoscape 3.2.1 software to construct a drug-target network to visualize the interactions between this systems (Fig. 1C).

Next, we performed enrichment analysis of the Kyoto Encyclopedia of Genes and Genomes (KEGG) molecular signaling pathways and disease types for the 142 drug targets that were obtained using DAVID v 6.8 and KOBAS 3.0 software, respectively. The results indicated that the above targets were significantly associated with “Pathways in cancer” and “Cancers” (Fig. 1D and E). Therefore, these prediction results not only help reveal the pharmacological mechanism of CR but also suggest that CR has great potential in cancer treatment.

### Oral administration of CR can inhibit the growth of CC cells in C57BL/6 mice

**Fig. 2:**
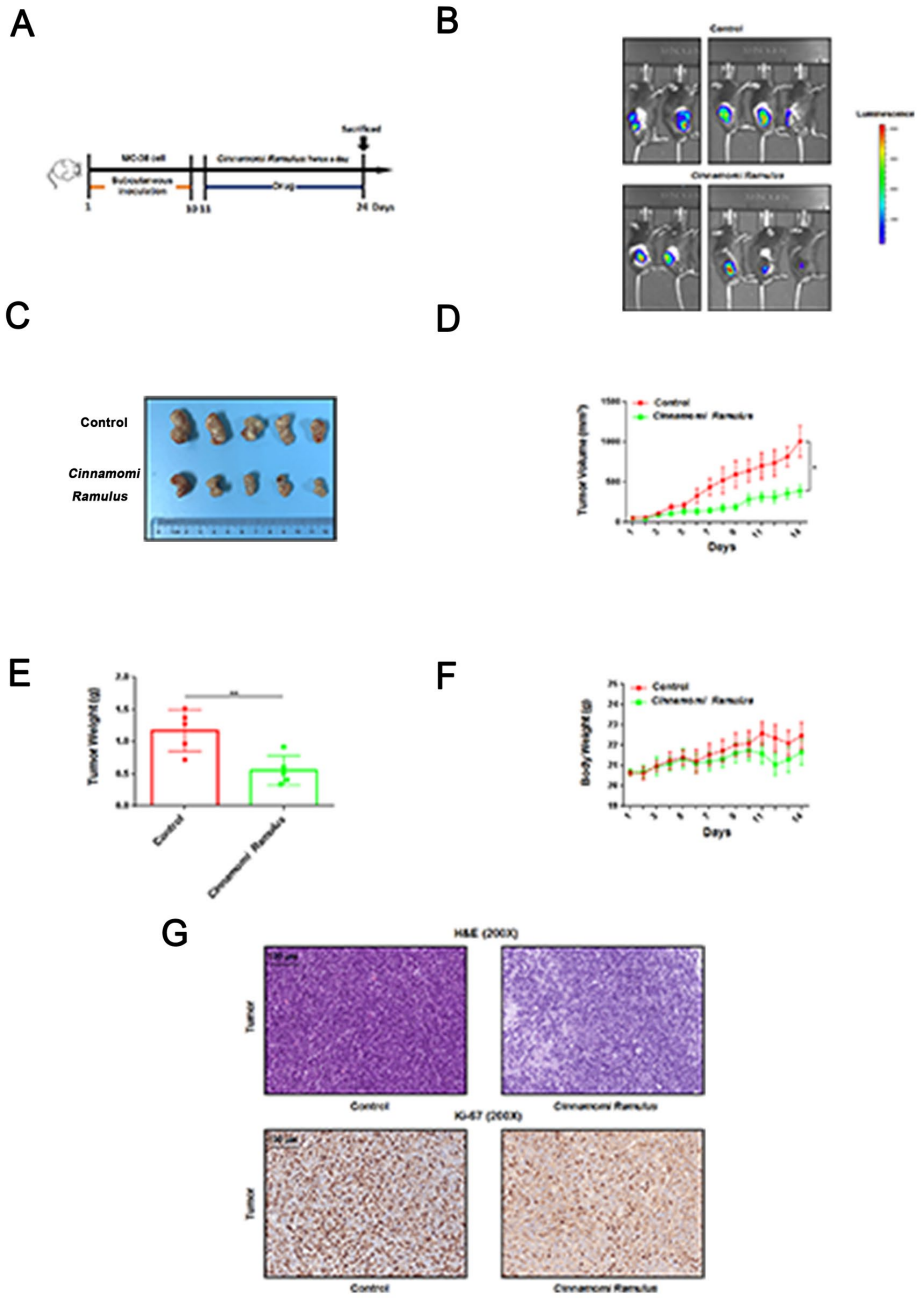
CR suppressed development of the xenografted CC tumors on C57BL/6 mice. **A** Workflow for this in vivo assay. **B** and **C** The photos of tumor sizes were shown on the day 14. **D** The tumor volumes were measured and calculated once daily for 14 consecutive days. **E** The tumors were resected and weighted on the day 14. **F** The body weight were measured once daily for 14 consecutive days. **G** H&E and immunohistochemistry staining for Ki-67 were performed by using the tumor slides from control and *CR*-treated groups. *p < 0.05 and **p < 0.01 based on the Student's *t*-test

To investigate the direct tumor suppressor effect of CR in vivo, we assessed its specific effects on xenograft tumors in C57BL/6 mice (subcutaneously injected with MC-38 cells) (Fig. 2A). As shown in Fig. 2B and C, compared with the control, the oral administration of CR significantly inhibited the growth of MC38 xenograft tumors in vivo. By day 14, the tumor volume in the CR treatment group was approximately 2.6 times that in the control group (*p* < 0.05) (Fig. 2D). Furthermore, there was a significant difference in tumor weight between these two groups (*p* < 0.05); however, there was no significant difference in the body weight of mice between these two groups (Fig. 2E and F). Next, through hematoxylin and eosin (H&E) tissue staining, we found that compared with the control group, tumor tissue in the CR treatment group showed obvious necrotic cells; the subsequent immunohistochemical analysis showed that the number of Ki-67-positive cells was significantly reduced in the CR treatment group, thus indicating that CR had an antiproliferative effect on colon tumor cells (Fig. 2G). Therefore, the above results provide convincing evidence that CR has direct anti-CC activity.

### CR can significantly inhibit the viability and motility of CC cells

**Fig. 3:**
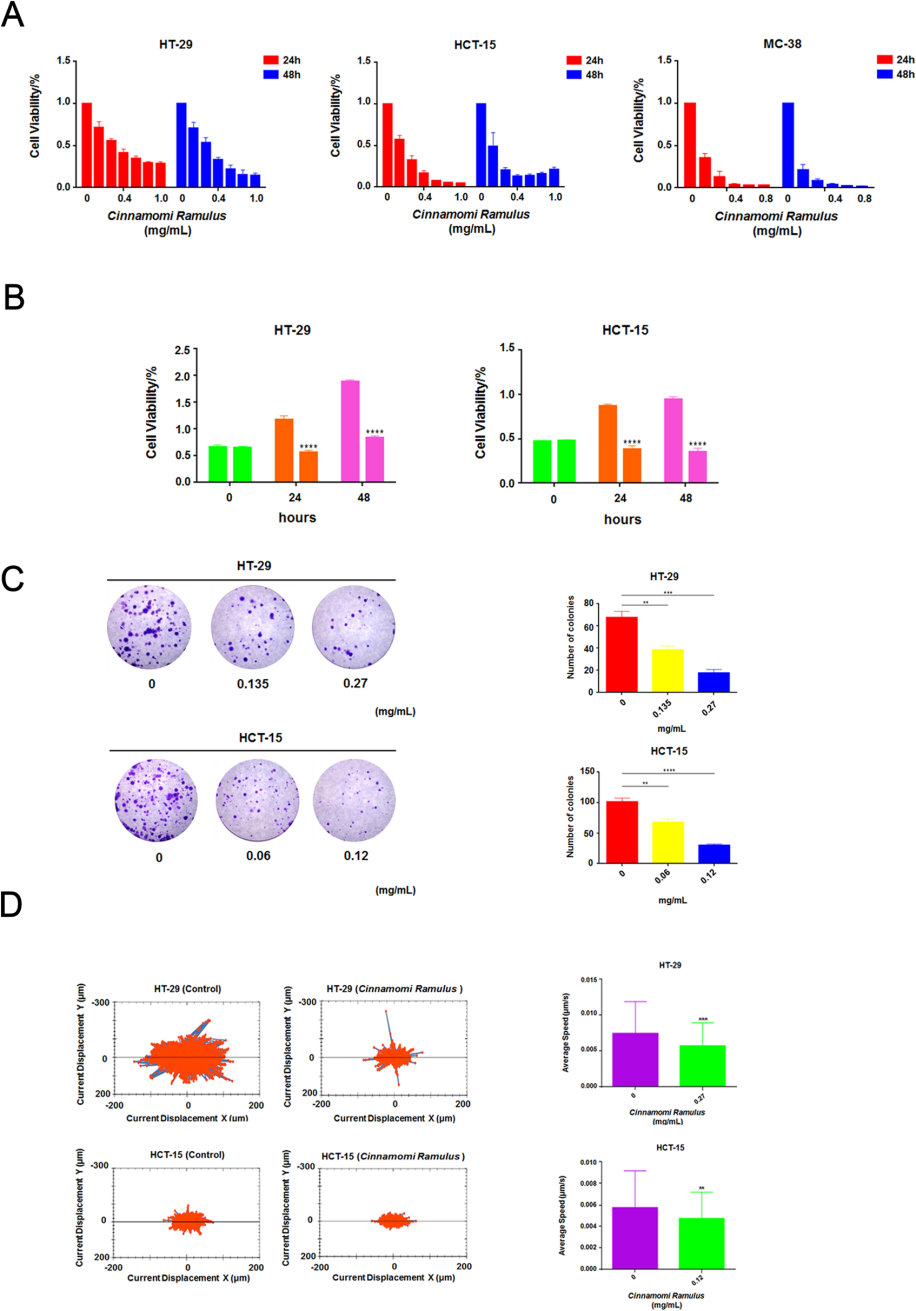
CR inhibited CC cells growth and decreased its viability. **A** CCK-8 assay was performed to measure cell viability at 24 h and 48 h after *CR* treatment at different dosages (0, 0.1, 0.2, 0.4, 0.6, 0.8, 1.0 mg/mL). **B** CCK-8 assay was performed to measure cell growth ability at 24 h and 48 h after *CR* treatment at 24 h IC_50_ (0.27 mg/mL and 0.12 mg/mL) in HT-29 and HCT-15 cells, respectively. **C** Cell colony formation assay was performed to measure cell clonality after *CR* treatment for 24 h. ***p < 0.001 and ****p < 0.0001 based on the Student's *t*-test. **D** The Operetta CLS High Content Analysis System was used to evaluate the accumulated distance of CC cells at 24 h after *CR* treatment. **p < 0.01 and ***p < 0.001 based on the Student's *t*-test

Subsequently, we performed a series of in vitro CR pharmacodynamics experiments using CC cells. The results of the CCK-8 assay showed that CR had a dose- and time-dependent inhibitory effect on the growth of HT-29, HCT-15 and MC-38 cells (Fig. 3A). The half maximal inhibitory concentration (IC_50_) analysis at 24 h showed that the IC_50_ values for CR in HT-29 and HCT-15 human colon carcinoma cells were 0.278 ± 0.019 mg/mL and 0.123 ± 0.014 mg/mL, respectively. The results of the subsequent cell growth experiments indicated that the viability of HT-29 and HCT-15 cells significantly decreased at 48 h compared with the control group (Fig. 3B). Meanwhile, the cell colony formation assay showed that the colony formation ability of the cells decreased in a dose-dependent manner after 24 h of treatment with CR (Fig. 3C). In addition, we also used a high-content microscope to track the motility of cells in real time. The average cumulative movement of the cells in the CR treatment group was less than the control group at 24 h, indicating that the cell viability in the CR treatment group was significantly inhibited (Fig. 3D). Therefore, the above results indicate that CR has a direct inhibitory effect on the survival and motility of CC cells.

### Construction of CR anti-CC PPI systematic network and core target enrichment analysis

**Fig. 4:**
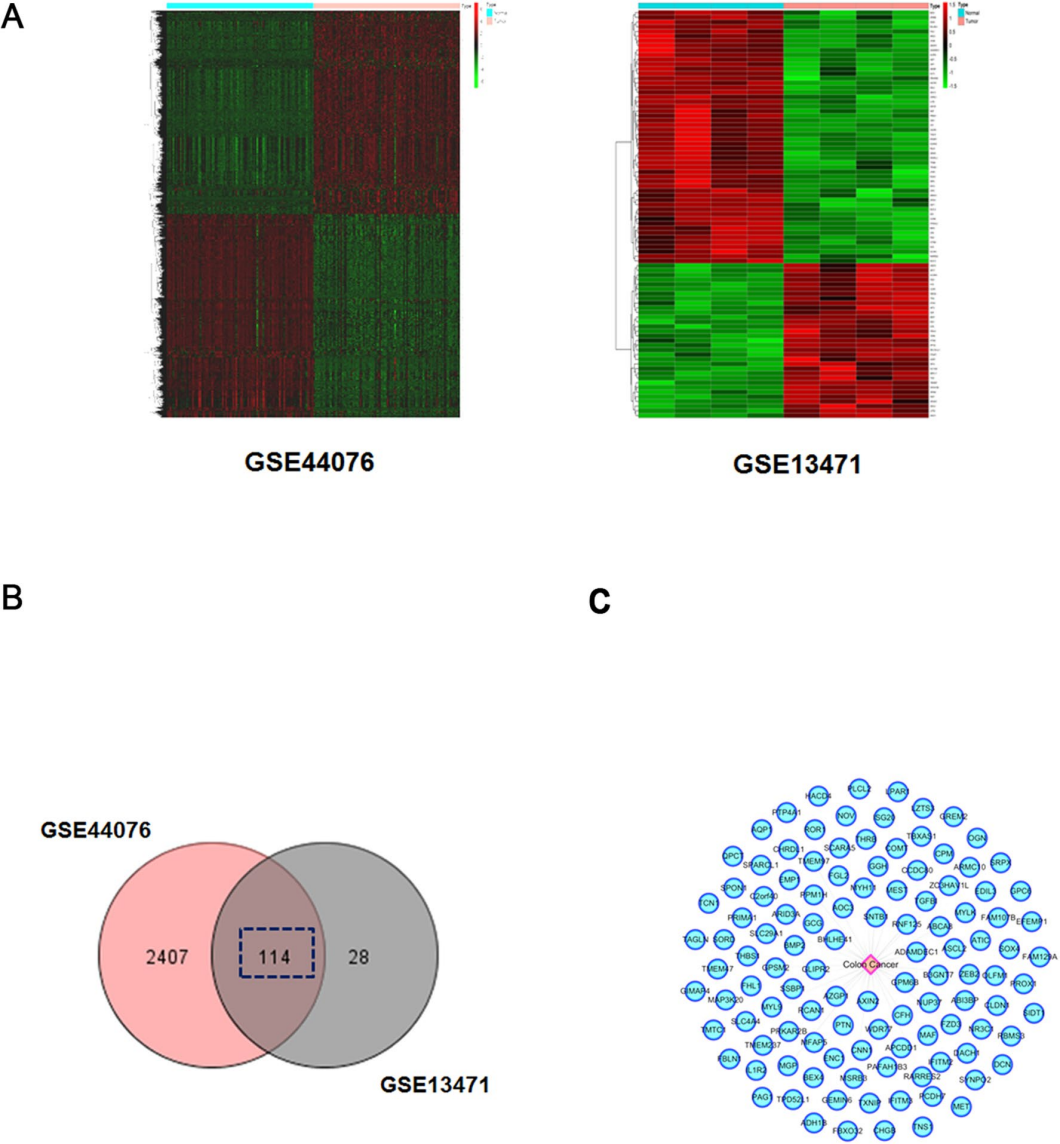
The known CC-related targets were screened from Gene Expression Omnibus (GEO) database. **A** Two heat maps from GEO chips, including GSE44076 and GSE13471. **B** The Venn diagram of 114 common CC-related targets from two GEO chips. **C** Construction of the CC-related targets systematic network

**Fig. 5:**
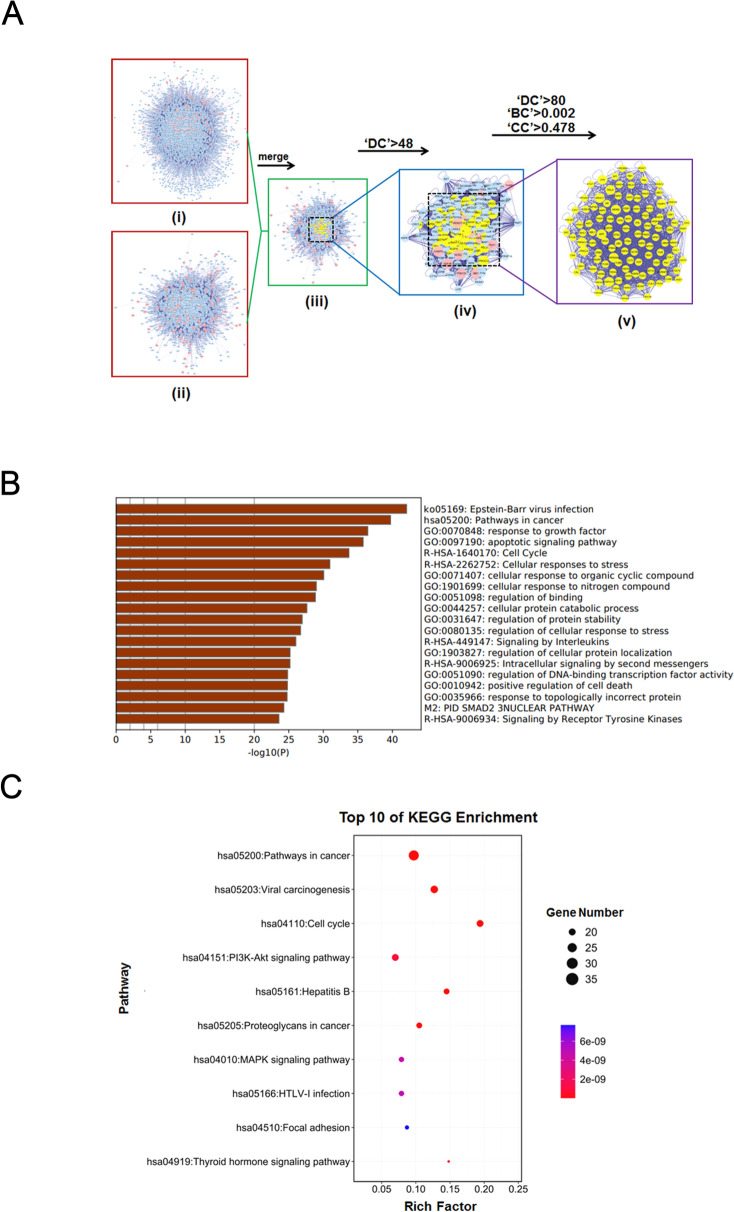
Screeening the candidate core targets for CR against CC and enrichment analyses. **A** (i) The PPI network of *CR* putative targets was made of 5575 nodes and 103,469 edges. (ii) The PPI network of CC-related targets was composed of 2027 nodes and 34,539 edges. (iii) The PPI network of *CR* against CC- related targets was made of 1181 nodes and 22,173 edges. (iv) The PPI network of key targets extracted from iii, in which 300 nodes and 8604 edges were shown. (v) The PPI network of candidate core targets extracted from iv, in which 111 nodes and 2312 edges were shown. **B** Candidate core targets were enriched in the representative biological processes (GO) by using Metascape (*p* < 0.05). **C** Candidate core targets were enriched in the representative signaling pathways (KEGG) by using DAVID v6.8 (*p* < 0.05)

With the help of network pharmacology and bioinformatics techniques, we used the Bisogenet plugin in Cytoscape 3.2.1 software to perform PPI analysis of the 142 potential drug therapeutic targets identified for CR. A total of 5,575 nodes and 103,469 edges were obtained. Then, through the GEO online database, the gene expression results of two microarrays of human CC and paired paracancerous tissues (GSE44076 and GSE13471) were downloaded and analyzed. After screening based on the conditions of *p* < 0.01 and fold change > 2, 114 relevant disease targets corresponding to CC were identified (Fig. 4A–C and Additional file 2: Table S2). Subsequently, the Bisogenet plugin was used to perform a PPI analysis of the above 114 disease targets. A total of 2027 nodes and 34,539 edges were obtained. Subsequently, to more accurately predict the potential core targets of CR for the treatment of CC, we used the CytoNCA plugin in Cytoscape software to systematically integrate the PPI analysis results for the drug targets and the disease targets. The conditions 'DC' > 48 and 'DC' > 80, 'BC' > 0.002, and 'CC' > 0.478 were used to perform core mining of the integrated network at the topology level, and 111 potential core targets were predicted (Fig. 5A and Additional file 3: Table S3).

To further predict the potential biological processes and related molecular mechanisms involving the abovementioned 111 core targets, we used Metascape and DAVID v6.8 to perform an enrichment analysis of the biological functions (GO-Biological Process, GO-BP) and molecular mechanisms (KEGG). The results of the GO-BP analysis indicated that the above targets were closely associated with processes such as “pathways in cancer”, “apoptotic signaling pathways”, and “cell cycle” (Fig. 5B), and the results of the KEGG analysis indicated that the above targets were closely associated with mechanisms such as ‘pathways in cancer’, ‘viral carcinogenesis’, the ‘cell cycle’, the ‘PI3K-Akt signaling pathway’, and the ‘MAPK signaling pathway’ (Fig. 5C). The results of these “virtual studies” provide clues for further revealing and verifying the pharmacological mechanism of CR in the treatment of CC.

### CR can induce apoptosis and G2/M phase arrest in CC cells

**Fig. 6:**
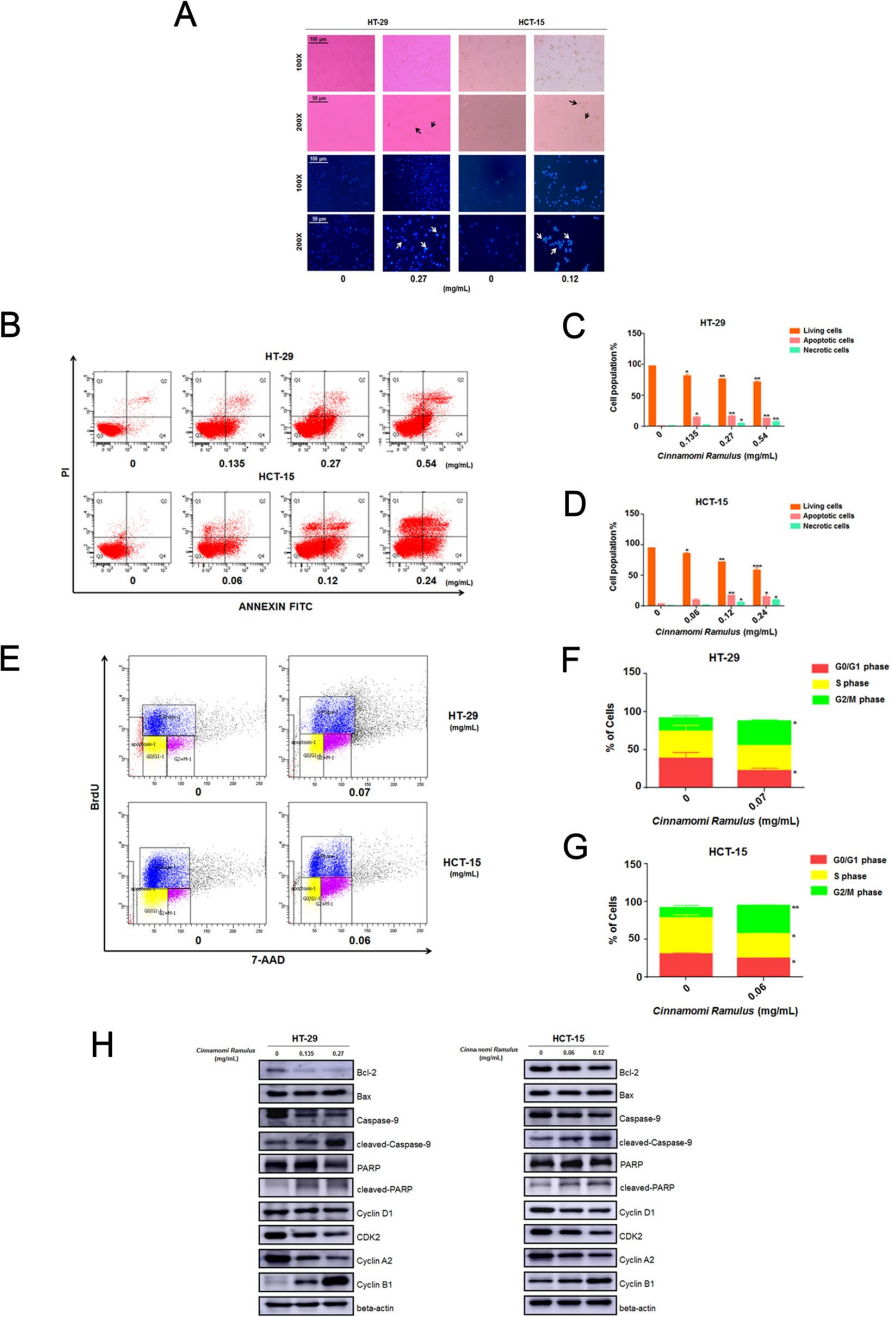
CR treatment resulted in apoptosis and disturbance of cell cycle progression in CC cells. **A** The cell morphology was observed in white light and fluorescence field using an inverted microscope. **B** Induction of apoptosis of HT-29 and HCT-15 cells after *CR* treatment for 24 h. The apoptotic events was assessed by flow cytometry. **C** and **D** Statistical analysis of the percentages of the apoptotic cells in HT-29 and HCT-15 cells. *p < 0.05, **p < 0.01 and ***p < 0.001 based on the Student's *t*-test. **E** Cell cycle analysis of HT-29 and HCT-15 cells following *CR* treatment for 24 h by flow cytometry. **F** and **G** Statistical analysis of the proportions of the cells at different phases in HT-29 and HCT-15 cells. *p < 0.05 and ***p < 0.001 based on the Student's *t*-test. **H** HT-29 and HCT-15 cells were treated with *CR* at different concentrations for 24 h. After proteins were extracted, the expression levels of Bcl-2, Bax, pro-caspase-9, cleaved-caspase-9, pro-PARP, cleaved-PARP, Cyclin D1, CDK2, CyclinA2 and CyclinB1 were analyzed by WB assay, respectively

Next, we conducted a series of cell function experiments to verify the results of the above enrichment analysis. First, compared with the control group, the cells after CR treatment showed typical characteristics of apoptosis, such as shrinkage, rounding, and loss of contrast. Meanwhile, fluorescence observation after Hoechst 33,342 staining revealed that nuclei after CR treatment exhibited dense staining or dense fragmented staining (Fig. 6A). In addition, the flow cytometry results indicated that the apoptotic cell population stained with Annexin-V FITC significantly increased in a dose-dependent manner after CR treatment (Fig. 6B–D). The WB results also indicated that CR promoted the accumulation (dose-dependent) of the proapoptotic protein Bax and cleaved caspase-9 and PARP and the downregulation of antiapoptotic protein Bcl-2 (Fig. 6H and Additional file 4: Fig. S1).

In addition, we also performed cell cycle analysis via BrdU incorporation and flow cytometry to evaluate the effect of CR on the cell cycle of CC cells. We used the 24 h IC_25_ concentrations of CR to HT-29 (0.07 mg/mL) and HCT-15 (0.06 mg/mL), respectively. The results showed that CR treatment arrested the cells at the G2/M phase, thereby significantly inhibiting their growth and proliferation (Fig. 6E–G). The WB results confirmed the accumulation of the G2/M phase-specific marker cyclin B1 and the downregulation of cyclin D1, CDK2 and cyclin A2 in CR-treated CC cells (Fig. 6H and Additional file 4: Fig. S1). In summary, the above results all indicated that CR inhibited the growth of CC cells by inducing apoptosis and cell cycle arrest.

### CR can inhibit the growth of CC cells by blocking the Akt/ERK signaling pathways

**Fig. 7:**
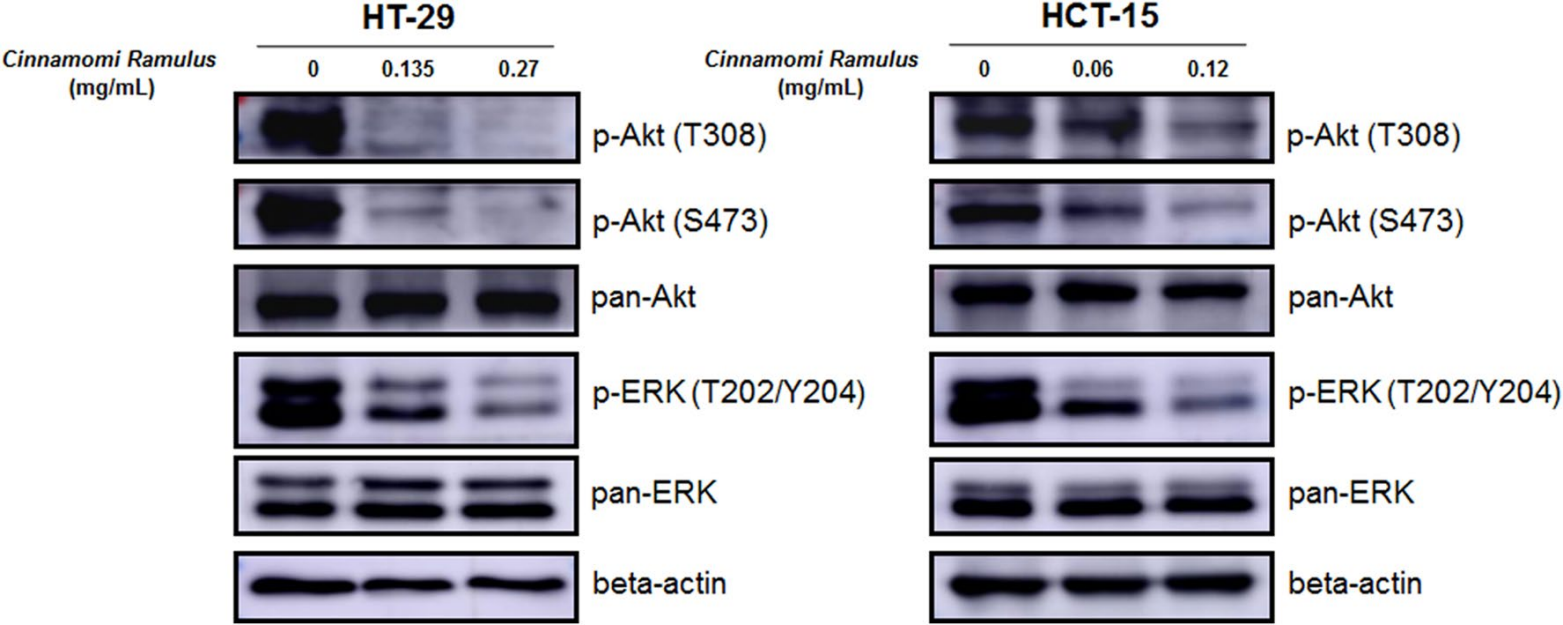
Down-regulating the phosphorylation protein expression level of Akt and ERK signaling pathways with CR treatment. HT-29 and HCT-15 cells were treated with *CR* at different concentrations for 24 h. After proteins were extracted, the expression levels of p-Akt (T308), p-Akt (S473), pan-Akt, p-ERK (T202/Y204) and pan-ERK were analyzed by WB assay, respectively

To further explore the potential molecular mechanism by which CR inhibits the growth of CC, we next examined the key signaling pathways involved in cell proliferation and viability. Among them, we selected the PI3K-Akt and MAPK signaling pathways based on the results of the KEGG pathway enrichment analysis. The WB results indicated that after CR treatment, the expression levels of key protein factors, such as p-Akt (T308 and S473) and p-ERK (T202/Y204), in the above pathways in the two CC cells were significantly inhibited (Fig. 7 and Additional file 5: Fig. S2). This result indicates that the effects of CR, i.e., inducing apoptosis and cell cycle arrest and inhibiting the growth of CC cells, may be the result of the simultaneous inhibition of the Akt/ERK signaling pathway, thus demonstrating once again that CHM has “multi-component, multi-target, and multi-function” pharmacological characteristics.

### Taxifolin is a potential key active component of CR in the treatment of CC

**Fig. 8:**
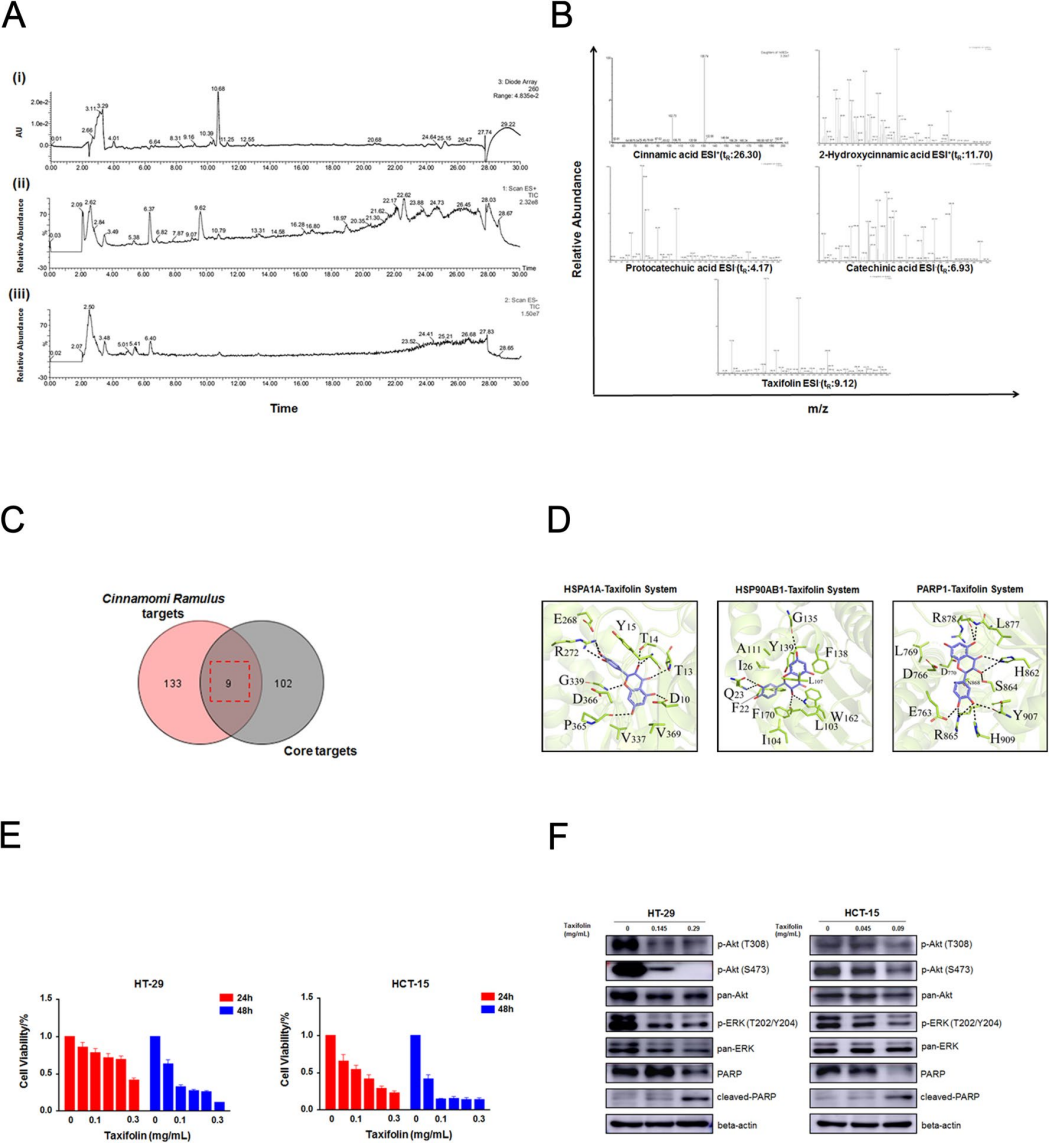
Five main ingredients obtained from *CR* by using UPLC-MS/MS and relevant analyses. **A** (i) The ultraviolet absorption wavelength map. (ii) The total ion chromatography (TICs) in positive ion modes. (iii) The total ion chromatography (TICs) in negative ion modes. **B** The five main ingredients were identified by secondary mass spectrometry. **C** The Venn diagram of 9 common key core targets from *CR-*related targets and candidate core targets. **D** Binding modes from AutoDock 4.2. Taxifolin with HSP1A1, HSP90AB1 and PARP1, respectively. **E** CCK-8 assay was performed to measure cell viability at 24 h and 48 h after taxifolin treatment at different dosages (0, 0.05, 0.1, 0.15, 0.2, 0.3 mg/mL). **F** HT-29 and HCT-15 cells were treated with taxifolin at different concentrations for 24 h. After proteins were extracted, the expression levels of pro-PARP, cleaved-PARP, p-Akt (T308), p-Akt (S473), pan-Akt, p-ERK (T202/Y204) and pan-ERK were analyzed by WB assay, respectively

Next, we used ultraperformance liquid chromatography-tandem mass spectrometry (UPLC–MS/MS) to identify the potential active components in CR. A total of 5 key components were identified from the crude extract of CR, i.e., cinnamic acid (t_R_: 26.30 min), 2-hydroxycinnamic acid (t_R_: 11.70 min), protocatechuic acid (t_R_: 4.17 min), catechinic acid (t_R_: 6.93 min), and taxifolin (t_R_: 9.12 min) (Fig. 8A and B and Table 2). Intriguingly, 3 of the 5 components detected above are potential active components that were previously screened based on ADME-related characteristics or literature searches, thus again confirming the reliability of the network pharmacology prediction method. To further explore the molecular mechanism by which CR affects CC at the protein level, we used AutoDock 4.2 software to perform molecular docking analysis between the above active ingredients and key targets. The key targets were obtained by combining the previously obtained 142 drug targets of CR with 111 core targets of CR for CC and identifying areas of overlap; ultimately, a total of 9 key targets were obtained (Fig. 8C). Next, we predicted the docking efficiency between the above 5 active ingredients and the 9 key targets. The results indicated that the binding affinities between taxifolin and the targets HSP1A1, HSP90AB1 and PARP1 (docking energies of − 9.25, − 9.17 and − 9.05 kcal/mol, respectively) were all higher than those between the remaining 4 components and other proteins (Fig. 8D and Table 3).

**Table 2:** Identification of main ingredients in CR by UPLC-MS/MS data

No	t_R_ (min)	Molecular formula	Selected ion	Theoretical	Experimental	MS/MS fragmentions	Compounds
1	26.30	C_9_H_8_O_2_	[M–H]^+^	148.16	149	130.74, 102.73	Cinnamic acid
2	11.70	C_9_H_8_O_3_	[M–H]^+^	164.16	165	119.37, 90.92	2-Hydroxycinnamic acid
3	4.17	C_7_H_6_O_4_	[M–H]^−^	154.12	153.51	109.14, 92.29, 80.73	Protocatechuic acid
4	6.93	C_15_H_14_O_6_	[M–H]^−^	290.27	289.03	244.85, 202.74, 109.09	Catechinic acid
5	9.12	C_15_H_12_O_7_	[M–H]^−^	304.25	302.85	192.91, 134.74	Taxifolin

**Table 3:** The results of molecular docking studies of five main ingredients in the active sites of nine proteins performed using AutoDock

Proteins	Ligands				
	Cinnamic acid	2-Hydroxycinnamic acid	Protocatechuic acid	Catechinic acid	Taxifolin
PRKCA	− 5.47	− 5.83	− 6.00	− 7.44	− 7.55
HSP90AB1	− 7.33	− 4.58	− 6.88	− 6.27	− 9.17
APP	− 5.68	− 5.61	− 5.09	− 7.52	− 8.04
RELA	− 7.09	− 6.72	− 6.52	− 7.10	− 7.05
JUN	− 4.20	− 4.66	− 3.98	− 4.71	− 5.21
MDM2	− 5.49	− 5.53	− 5.13	− 6.71	− 6.83
PRKACA	− 7.09	− 6.81	− 6.00	− 7.93	− 8.19
HSPA1A	− 6.24	− 5.52	− 5.39	− 7.56	− 9.25
PARP1	− 6.79	− 6.62	− 6.41	− 8.62	− 9.05

(kcal/mol)

Next, in order to explore the effect of taxifolin on CC, we have further verified. Firstly, the CCK-8 assay results showed that taxifolin, as a potential key active component of CR, had a dose- and time-dependent inhibitory effect on the growth of human CC HT-29 and HCT-15 cells (Fig. 8E). Meanwhile, the 24 h IC_50_ values for taxifolin in HT-29 and HCT-15 were 0.294 ± 0.005 mg/mL and 0.099 ± 0.010 mg/mL, respectively. Then, the WB results indicated that after taxifolin treatment, the expression levels of key protein factors in Akt/ERK pathway, such as p-Akt (T308 and S473) and p-ERK (T202/Y204), were significantly inhibited in HT-29 and HCT-15 cells (Fig. 8F and Additional file 6: Fig. S3). Furthermore, taxifolin could also promote the accumulation of the cleaved PARP, which was the potential key target of taxifolin (Fig. 8F and Additional file 6: Fig. S3). In fact, the relationship between PARP1 and Akt/ERK signaling is also very close. For instance, a study indicated that luteolin promoted the activation of PARP1 via inhibition the expression and phosphorylation of Akt and ERK [20]. Taxifolin is very likely to be a potential key active component of CR in the treatment of CC; however, more systematic verification is still needed.

## Discussion

Network pharmacology is an emerging discipline that studies the occurrence and development of diseases from the perspective of biological networks and investigates the mechanism of interaction between drugs and diseases, thereby guiding the development of new drugs. The study of the relationship between TCM and disease addresses holistic, synergistic and dynamic components, coinciding with the TCM perspectives of “holistic view” and “dialectical therapy” [21]. Network pharmacology can elucidate the effect of drugs on biological networks from the perspective of macroscopic or overall regulation and provide novel research ideas and technical means for studying the mechanism of action of CHM [22]. In recent years, we have carried out a series of basic studies using this advanced technology [10, 18, 23, 24]. In this study, we first identified 14 potential active compounds in CR from the TCMSP database and related literature and explored the potential protein targets corresponding to these components. We also performed enrichment analysis of these targets. The results indicated that CR may play a role in the treatment of cancer. Next, given that the pharmacological mechanism of action of CR in the treatment of CC is currently unknown and the potential clinical application of CR has yet to be fully evaluated, we constructed a PPI systematic network for CR treatment of CC at the target level and identified 111 potential core targets. We further analyzed the potential molecular mechanism of CR action in the treatment of CC. Finally, a series of in vivo and in vitro experiments confirmed that CR could inhibit the growth of CC cells by negatively regulating the Akt/ERK signaling pathway, suggesting that CR has certain potential in the treatment of CC.

In this study, our results showed that CR could induce apoptosis and cell cycle arrest in CC cells by inhibiting Akt/ERK signaling pathways. We further provided the evidence of significant changes of the key factors involved in these pathways upon CR treatment by western blotting. In fact, mounting evidence has shown that inhibition of Akt/ERK pathways can lead to cell cycle arrest or apoptosis [25–30]. For example, a recent study showed that inhibition of Akt/ERK pathways by *Hedyotis diffusa Willd* (HDW) extracts could induce apoptosis in glioblastoma [25]. Likewise, inactivation of Akt and ERK signaling pathways by Harmine resulted in cell cycle arrest and mitochondrial pathway-mediated cellular apoptosis in colon cancer cells [30].

Next, we identified 5 potentially active monomers in CR via UPLC–MS/MS: cinnamic acid, 2-hydroxycinnamic acid, protocatechuic acid, catechinic acid, and taxifolin. Interestingly, previous studies have found that some of the above components have potential antitumor effects. For example, cinnamic acid, as a key component in CR, can induce apoptosis in melanoma cells and reduce their proliferation rate, and its derivatives can also induce apoptosis in CC and cervical cancer cells [31, 32]. Protocatechuic acid can inhibit the formation of melanin in melanoma cells; additionally, its combined application with 5-fluorouracil has been shown to promote antiproliferative and proapoptotic effects by inhibiting the colony formation ability of gastric cancer AGS cells [33, 34]. Catechin compounds mainly include catechinic acid, epicatechin, epigallocatechin, epicatechin gallate, and epigallocatechin gallate. Relevant studies have found that catechinic acid can increase the sensitivity of ovarian cancer cells to platinum-based drugs, and can damage DNA and reduce DNA synthesis to inhibit the proliferative activity of tumor cells [35].

In addition, the results of the molecular docking showed that among the 45 docking systems, the top 3 binding systems involved the interactions between taxifolin and HSPA1A, HSP90AB1 and PARP1. Taxifolin, also known as dihydroquercetin, is a flavonoid. In recent years, the results of several studies have shown that taxifolin has an inhibitory effect on many types of tumors. For example, taxifolin inhibits diacylglycerol acyltransferase (DGAT) and microsomal triglyceride transfer protein (MTP) activity in human hepatocellular carcinoma (HepG2) cells, thus exerting an antihepatocarcinogenic effect; it also has a significant inhibitory effect on the colony-forming ability of HCT-116 human colon cancer cells [36, 37]. Additionally, the abovementioned targets are also closely related to cancers. As a molecular chaperone, the expression level of heat shock protein 90 (Hsp90) in tumor cells is approximately 2–10 times that in normal cells. It has been reported that the expression of Hsp90 and its client proteins (e.g., EGFR, AKT, Raf-1, HER2, and CDK4/6), which are closely related to the development and progression of tumors, is increased [38] and that the inhibition of Hsp90 function leads to the degradation of tumor-associated client proteins [39]. Therefore, Hsp90 is a promising antitumor therapeutic target. A recent study found that the small molecule compound DCZ5248 is a novel dual inhibitor of Hsp90 and autophagy and has the potential to treat CC [40]. Interestingly, related studies also showed that taxifolin can inhibit the expression of HSP90, thereby significantly reducing the growth and proliferation activity of breast cancer cells [41]. There are approximately 17 members of the PARP family, and PARP1 is the most intensively studied member because of its broad application. Nosho et al. found that PARP1 was highly expressed in 70.3% of colorectal cancer tissues and that its expression level was correlated with tumor size and tumor differentiation. Another study also found that PARP1 was highly expressed in 68.2% of colorectal cancer tissues and closely associated with tumor location [42, 43], indicating that high PARP1 expression may be an important molecular event in colorectal cancer. A study showed that protocatechuic acid could induce the apoptosis of ovarian cancer cells and inhibit their growth by increasing the expression of PARP protein in spliceosomes [44]. Our WB results also showed that CR promoted the cleavage of PARP, thus inducing CC cell apoptosis. In summary, these results all support that CR shows potential as a multi-component, multi-target, and multi-pathway intervention option for the treatment of CC.

## Conclusions

Our results show that CR can regulate the key biological functions and processes related to the growth and viability of CC cells, such as apoptosis and cell cycle. The interference of these may be attributed to the simultaneous inhibition of multiple signaling pathways, including Akt and ERK pathways. In addition, further analyses also indicate that taxifolin may play an important role in the transmission of pharmacological activities, and HSPA1A, HSP90AB1 and PARP1 are expected to become potential therapeutic targets of CR against CC (Additional file 7: Fig. S4).

## Supplementary Information

**Additional file 1: Table S1.** The candidate drug targets of CR.

**Additional file 2: Table S2.** The CC-related targets from GEO.

**Additional file 3: Table S3. **The potential core targets.

**Additional file 4: Figure S1.** The original WB images for Bcl-2, Bax, pro-caspase-9, cleaved-caspase-9, pro-PARP, cleaved-PARP, Cyclin D1, CDK2, CyclinA2, CyclinB1 and beta-actin.

**Additional file 5: Figure S2.** The original WB images for p-Akt (T308), p-Akt (S473), pan-Akt, p-ERK (T202/Y204), pan-ERK and beta-actin.

**Additional file 6: Figure S3.** The original WB images for pro-PARP, cleaved-PARP, p-Akt (T308), p-Akt (S473), pan-Akt, p-ERK (T202/Y204), pan-ERK and beta-actin.

**Additional file 7: Figure S4.** The workflow of this study.

## Data Availability

The datasets used and/or analyzed during the current study are available from the corresponding author on reasonable request.

## Abbreviations

CR: *Cinnamomi Ramulus*
CC: Colon cancer
TCM: Traditional Chinese medicine
CHM: Chinese herb medicine
PPI: Protein–protein interaction
UPLC-MS/MS: Ultraperformance liquid chromatography-tandem mass spectrometry
DC: Degree centrality
BC: Betweenness centrality
CC': Closeness centrality
