# Supplementary figures and images for: Cinnamomi Ramulus inhibits the growth of colon cancer cells via Akt/ERK signaling pathways
Source: Chin Med. 2022 Mar 9;17:36. doi: 10.1186/s13020-022-00588-6 (PMC8905814; doi:10.1186/s13020-022-00588-6)

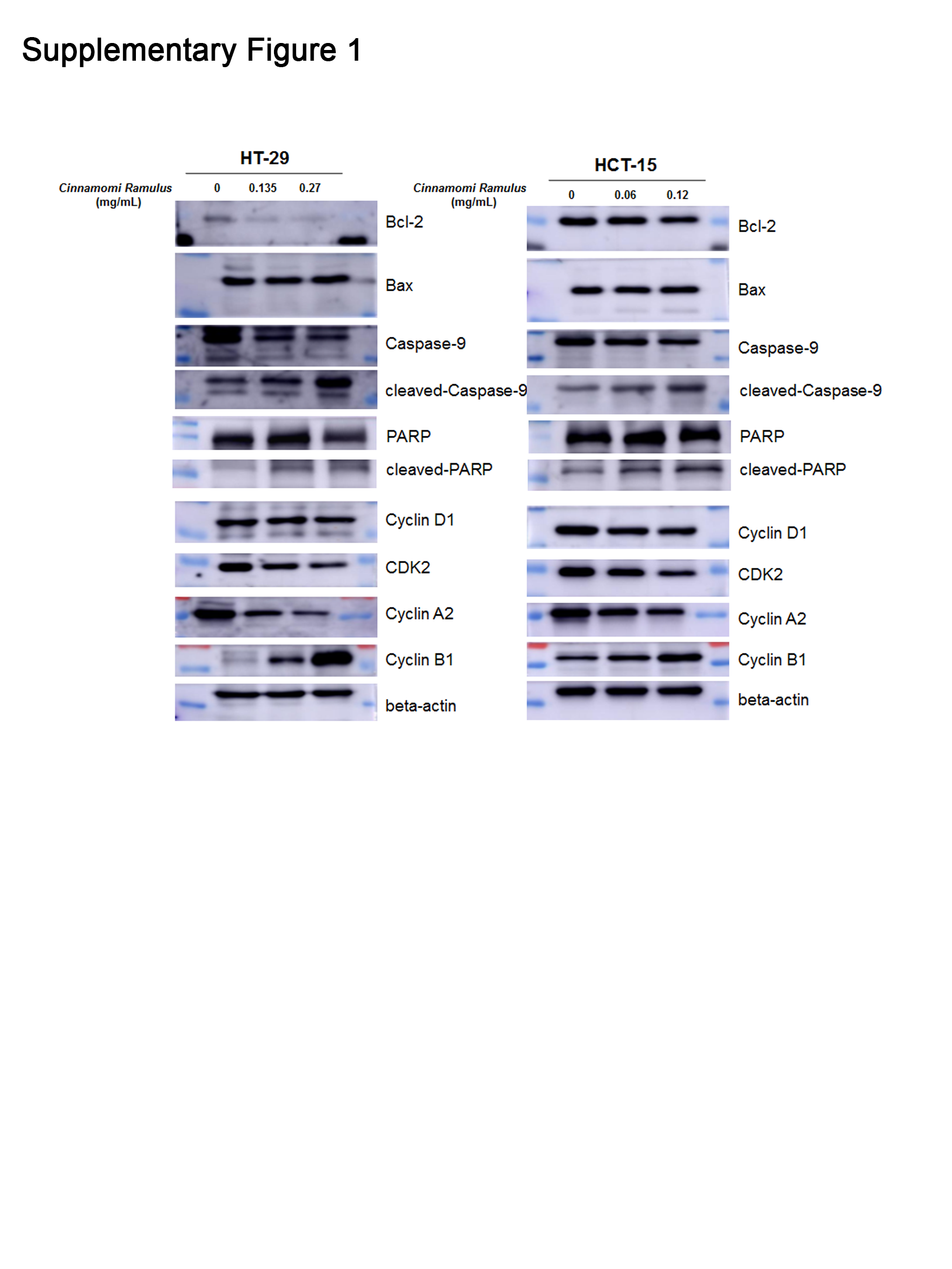

Supplement: Supplementary file 4 — Additional file 4: Figure S1. The original WB images for Bcl-2, Bax, pro-caspase-9, cleaved-caspase-9, pro-PARP, cleaved-PARP, Cyclin D1, CDK2, CyclinA2, CyclinB1 and beta-actin. [file 13020_2022_588_MOESM4_ESM.tif]

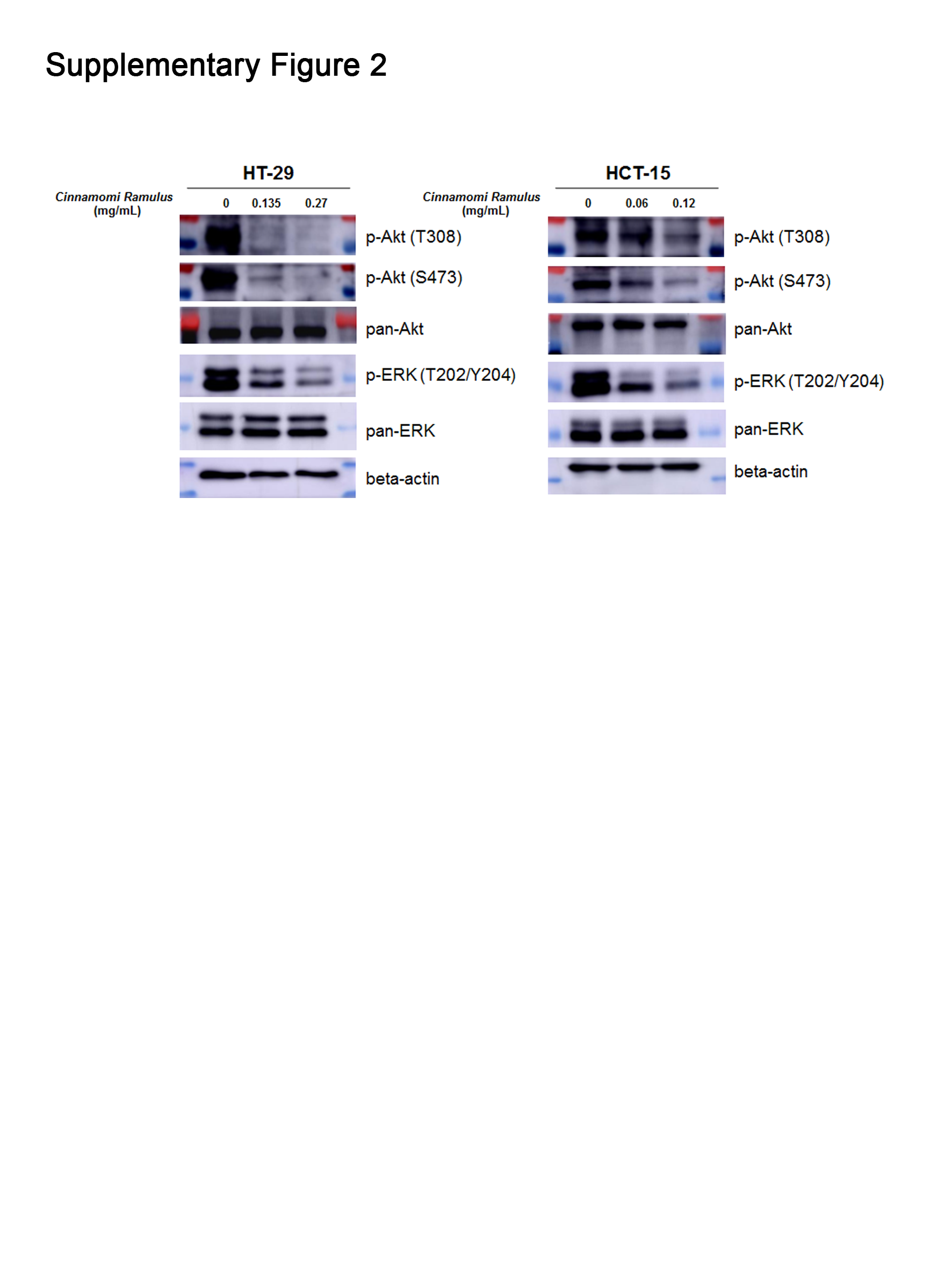

Supplement: Supplementary file 5 — Additional file 5: Figure S2. The original WB images for p-Akt (T308), p-Akt (S473), pan-Akt, p-ERK (T202/Y204), pan-ERK and beta-actin. [file 13020_2022_588_MOESM5_ESM.tif]

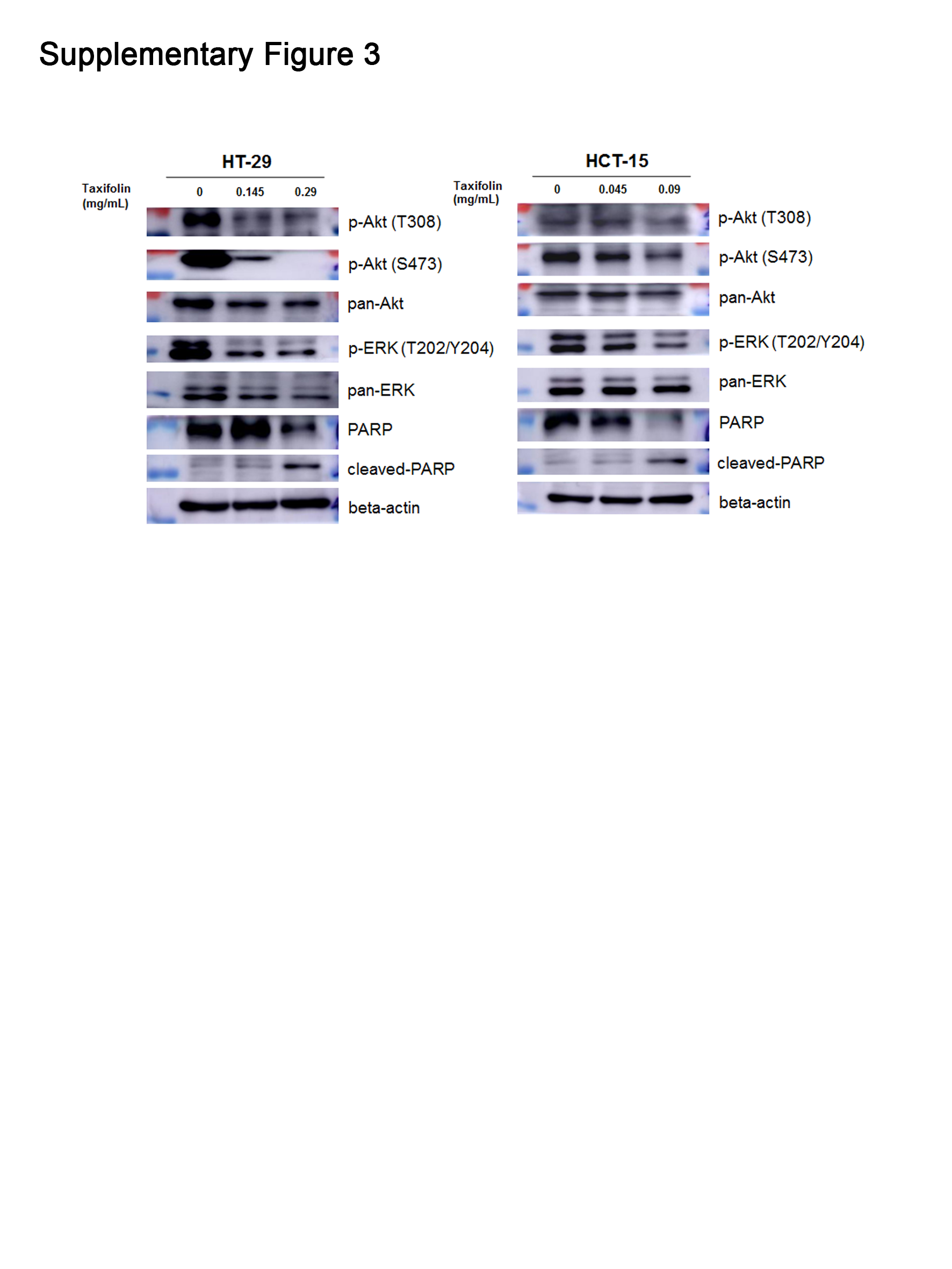

Supplement: Supplementary file 6 — Additional file 6: Figure S3. The original WB images for pro-PARP, cleaved-PARP, p-Akt (T308), p-Akt (S473), pan-Akt, p-ERK (T202/Y204), pan-ERK and beta-actin. [file 13020_2022_588_MOESM6_ESM.tif]

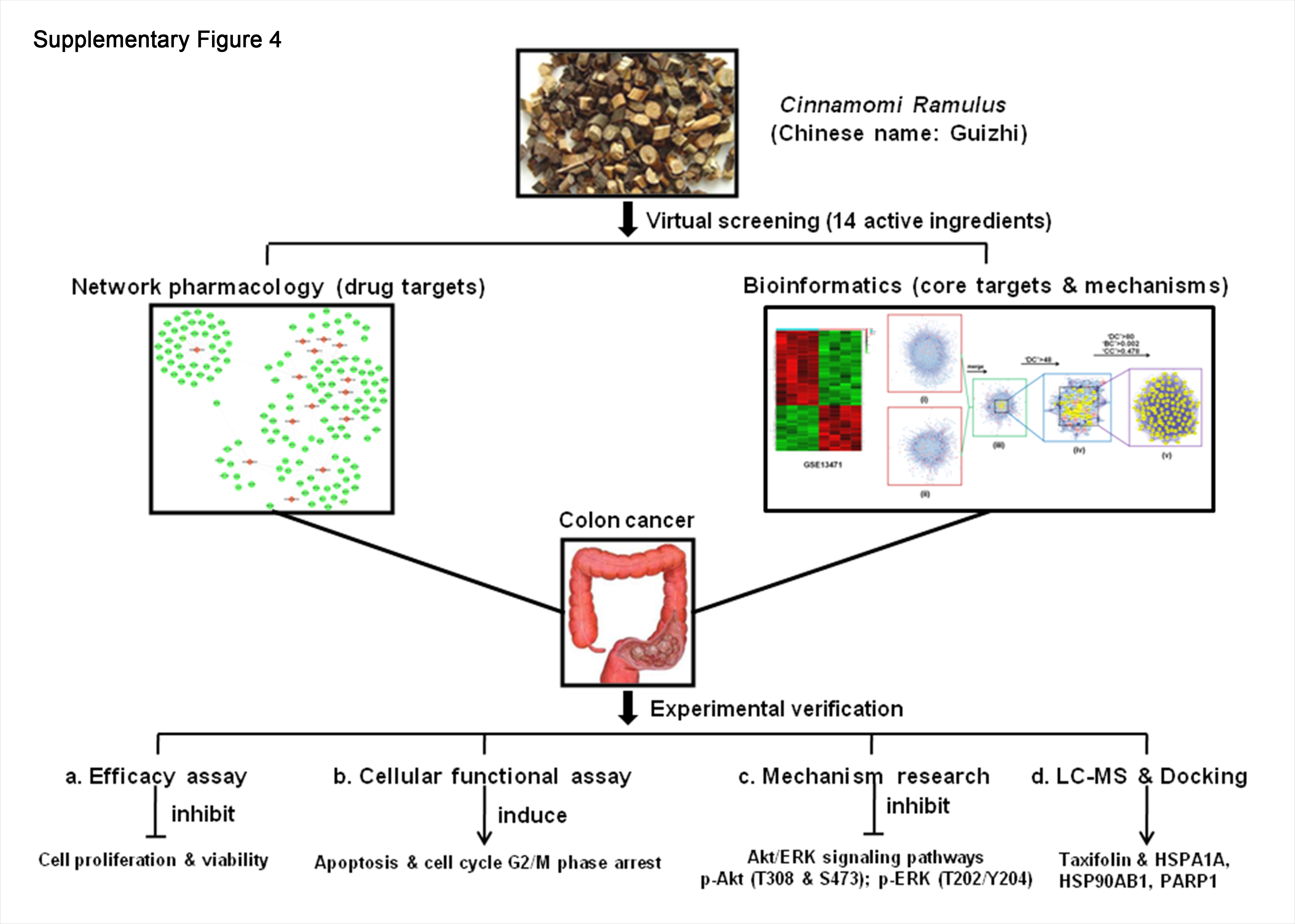

Supplement: Supplementary file 7 — Additional file 7: Figure S4. The workflow of this study. [file 13020_2022_588_MOESM7_ESM.tif]
